# The feeding preference and bite response between *Microtus fortis* and *Broussonetia papyrifera*

**DOI:** 10.3389/fpls.2024.1361311

**Published:** 2024-09-09

**Authors:** Shuangye Wang, Zihao Chen, Mengxin Wang, Meiwen Zhang, Chen Zhang, Tian Huang, Yunlin Zhao, Zhenggang Xu

**Affiliations:** ^1^ School of Basic Medicine, Guiyang Healthcare Vocational University, Guiyang, Guizhou, China; ^2^ Hunan Research Center of Engineering Technology for Utilization of Environmental and Resources Plant, Central South University of Forestry and Technology, Changsha, Hunan, China; ^3^ Key Laboratory of National Forestry and Grassland Administration on Management of Western Forest Bio-Disaster, College of Forestry, Northwest A & F University, Yangling, Shaanxi, China; ^4^ Dongting Lake Station for Wetland Ecosystem Research, Institute of Subtropical Agriculture, The Chinese Academy of Sciences, Changsha, Hunan, China; ^5^ Hunan Engineering Research Center of Ecological Environment lntelligent Monitoring and Disaster Prevention and Mitigation Technology in Dongting Lake Region, Hunan City University, Yiyang, Hunan, China

**Keywords:** rodent management, paper mulberry, botanical pesticides, plant secondary metabolites, defensive substance

## Abstract

**Introduction:**

*Broussonetia papyrifera* is a dioecious plant that is rich in various metabolites and widely distribute in Asia. *Microtus fortis* is a rodent that often causes damage to crops, especially in the Dongting Lake region of China. There is a wide overlap in the distribution areas for the above species and the *M. fortis* feeds on the leaves of the *B. papyrifera*. Preliminary experiments have shown that the reproduction of *M. fortis* is inhibited after feeding on the leaves of the *B. papyrifera*.

**Methods:**

In order to explore the potential of using *B. papyrifera* to develop botanical pesticides, we investigated the palatability and reactive substances. The feeding frequency of *M. fortis* on *B. papyrifera* leaves to that of on daily fodder and *Carex brevicuspis* that is the primary food for the wild population were compared. We also attempted to identify the responsive substances in *B. papyrifera* leaves that were bitten by *M. fortis* using metabolome analysis.

**Results:**

In general, *B. papyrifera* leaves exhibited a stronger attraction to *M. fortis*. *M. fortis* foraged *B. papyrifera* leaves more frequently, and the intake was higher than that of the other two. Differential metabolites were screened by comparing normal leaves and leaves bitten by *M. fortis*, meanwhile with the intervention of clipped leaves. A total of 269 substances were screened, and many of these were involved in the biosynthesis of secondary metabolites, including terpenoids and alkaloids. These substances may be related to the defense mechanism of *B. papyrifera* against herbivores.

**Discussion:**

These findings support further research examining animal–plant interactions and simultaneously provide insights into the utilisation of *B. papyrifera* resources and the management of rodents. The good palatability and the defense of *B. papyrifera* leaves suggest that they have the potential to contribute in development of plant rodenticide.

## 1 Introduction

Interactions between plants and herbivores, which play a significant role in shaping ecosystem structure and function through food web and nutrient cycles, are important ecological processes in natural evolution (Gong and Zhang, 2014). When attacked by herbivores, plants use various defensive measures, including chemical resistance traits. Research has shown that over 50000 plant secondary metabolites (PSMs) are associated with plant resistance to feeding (Gong and Zhang, 2014). However, these substances are metabolically expensive to synthesize and do not contribute to growth. In fact, the wounding events that plants suffer in nature are not exclusively caused by feeding from herbivores but also by a myriad of abiotic factors such as hail and wind (Fürstenberg-Hägg et al., 2013). Previous studies have indicated that plant reactions to herbivory feeding are different from those to mechanical damage (Kallure et al., 2022). Thus, plants always face the dilemma of balancing growth and development with defense, which has led to them developing the capacity to distinguish herbivore damage from mechanical wounding. “Plant immunity,” by which plants can induce resistance to an attacker, is a widespread phenomenon. Scholars revealed different herbivores can elicit distinct defense responses in plants based on the specific signals released by herbivores (Bingham and Agrawal, 2010; Xu et al., 2015a). One vital mechanism is that plants can perceive specific herbivores by recognizing their oral secretions, which elicit more intense volatile responses than mechanical damage alone (Arimura et al., 2004; Acevedo et al., 2015). In recent years, PSMs have been used as alternatives to synthetic pesticides to mitigate the challenge of herbivore sustainably, restrict the usage of synthetic pesticides, and promote the usage of environment-friendly options (Divekar et al., 2022). Until now, the study of the relationship between herbivores and plants mainly focuses on insects and plants, with less attention paid to mammals and plants. Rodents are the most diverse mammal species, and plants are the main food source for most of them (Kay and Hoekstra, 2008). This has negatively affected agricultural production (He et al., 2024). The PSMs produced by higher plants have generated many efforts to exploit their potential for rodent control (Hansen et al., 2016).

*Microtus fortis* is mainly distributed in more than 17 provinces in China, some areas of Russia, and in North Korea and Mongolia close to the northeast borderlands of China (Jiang et al., 2012). The subspecies of *M. fortis* that is distributed around the Dongting Lake wetland in China is a typical harmful rodent (Zhang et al., 2013). Previous studies showed that rodents would alter their food composition in different habitats and that *Carex brevicuspis* is the primary food for both adult and infant *M. fortis* in the Dongting Lake region (Wu et al., 1998; Guo et al., 1999; Yong et al., 2012). Tannins, proteins, and cellulose have been verified as important factors affecting the feeding choice of *M. fortis*, and protein content influences the weight of infant voles significantly (Xing et al., 2010). During the dry season, *M. fortis* primarily lives on the beaches of the Dongting Lake, which is rich in *C. brevicuspis*; however, when the water level of the lake rises during the flood season, the voles are forced to migrate outside the lake to scavenge crops, and they typically cause damage (Xu et al., 2015b). Rodent-proof walls have been built around Dongting Lake wetland to avoid consequent damage and restrain *M. fortis* population increase (Zhang et al., 2012). During break-out periods, the voles are usually killed using artificial behaviors or chemical rodenticides (Ye et al., 2006); each methods has advantages and disadvantages. The lethal chemical rodenticides such as anticoagulants remain the most effective tool for rodent pest management, and second-generation anticoagulants are used for rodent pest management in China due to their high toxicity and chronic mode of action (Ma et al., 2019). However, many highly toxic compounds on the market widely used in controlling rodents are also broad-spectrum toxicants that can kill non-target species (Campbell et al., 2015; Ruiz-Suárez et al., 2014). These toxicants have long persistence times in live animals and carcasses, which can cause the direct and indirect poisoning to humans, rodent predators, raptors and general scavengers (Bronstein et al., 2008; Valchev et al., 2008). Thus, using chemical rodenticides with high toxicity poses potentially significant environmental risks through the accumulation of the compound in the food chain (Alomar et al., 2018). Therefore, the development of affiliative rodent rodenticides, such as inhibitors from plants, is an urgent need.

*Broussonetia papyrifera* is a deciduous tree belonging to the Moraceae family that is fast-growing and highly adaptable (Yalley et al., 2020). These trees widely grow, and are planted in China, and the species is an important economic plant resource (Xi et al., 2013; Han et al., 2016). The leaves of artificially planted hybrid *B. papyrifera*, which contain less anti-digestive factor than the wild *B. papyrifera* leaves, have long been used as livestock feed (Zhang et al., 2009) to promote livestock growth (Kandylis et al., 2009; Tao et al., 2020). Additionally, PSMs abundant in *B. papyrifera* leaves exhibit various active effects (Hong et al., 2013), and five compounds that could potentially inhibit oestrogen biosynthesis in human ovarian granulosa were extracted and isolated from *B. papyrifera* leaves using methanol extraction. This suggests that substances in *B. papyrifera* leaves exert bidirectional effects on animals and are potential botanical rodenticides. *B. papyrifera* leaves could restrain rodent population increases so that they can be managed sustainably.

During our seasonal investigation around the Dongting Lake wetland, we found that *M. fortis* feeds on the wild *B. papyrifera* leaves during migration. Through laboratory experiments, we further found that the reproduction of *M. fortis* was inhibited after feeding on the leaves of *B. papyrifera* (Wang et al., 2023). Therefore, the present study focused on the feeding preference of *M. fortis* towards *B. papyrifera* and the PSMs changes in the leaves after feeding to understand the potency of *B. papyrifera* as a botanical rodenticide.

## 2 Materials and methods

### 2.1 Feeding experiment design

**Figure 1:**
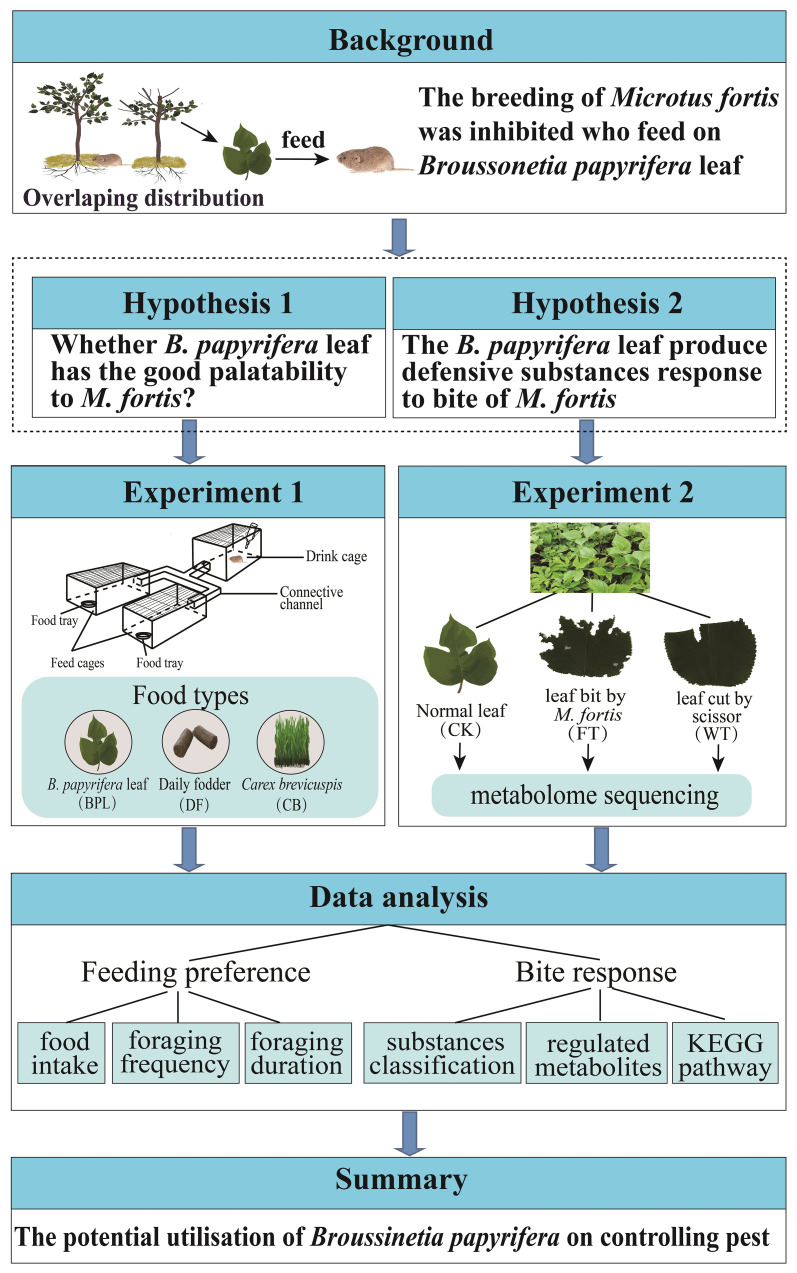
The research framework and methods in the study.

In order to confirm whether the *M. fortis* prefers to eat *B. papyrifera*, a free feeding experiment was conducted in the study. The free feeding equipment consists of three cages and channels (**Figure 1**). In the study, three types of food were selected for conducting relevant experiments, and they are basal fodder (BF), fresh *B. papyrifera* leaves (BPL) and fresh *C. brevicuspis* leaves (CB) respectively. Five feeding comparison models were tested under the same environmental conditions. For each model, the only difference was the kind of food in the trays. The experimental models included (1) the blank control model where the BF was added to two trays, (2) the BPL smell vs CB smell model where BPL and CB were ground and placed into bottles with holes, the bottles were placed in different cages, and BF was also placed into two trays to allow the voles to smell BPL and CB but feed on BF only, (3) the BPL vs BF model where BPL and BF were respectively placed into two trays, (4) the CB vs BF model where CB and BF were respectively placed into two trays, and (5) the BPL vs CB model where BPL and CB were respectively placed into two trays (**Supplementary Figure S1**). Four male and four female voles in total were fixedly used during feeding experiment. Each vole lived independently in a cage with free food and water, and was observed in each model twice. To avoid human interference, the feeding behaviors of voles were recorded using a camera, which was 1 m above the apparatus that allowed the full view. The vole individual was without eating for more than 5 hours before the test. The foods were placed in different trays according to models. Before recording the videos, the total weights of the food and trays were recorded. The videoing process lasts for 90 minutes and the feeding characteristic were calculated, including food intake, foraging frequency and foraging duration. The tray and food combined was weighed before and after each video to calculate food consumption. Videos were used to record the foraging behavior of the voles, including foraging frequency and duration. When the voles jumped into the feed cages from the channel and sustained for more than 5 s, they touched the food or the tray, and this was recorded as effective foraging.

The *M. fortis* used in the present study were the offspring of wild-caught individuals captured from the Dongting Lake area and maintained in the laboratory as outbred stock. The voles did not feed on any other food except for BF purchased from Hunan SJA Laboratory Animal Co., Ltd (http://www.hnsja.com/). The BPL were collected from the campus of Central South University of Forestry and Technology in Changsha, China (28°6′25.48′′ N, 112°59′37.68′′ E) where the *B. papyrifera* naturally grow with healthy soil. Meanwhile, CB also were collected from the east Dongting Lake wetland (28°30′-29°31′ N,110°40′-113°10′). The animal study protocol was approved by the Ethics Committee of the Institute of Subtropical Agriculture of the Chinese Academy of Sciences.

### 2.2 Analysis of *B. papyrifera* leaves responses to *M. fortis* bites

In order to determine the material response of *B. papyrifera* leaves to *M. fortis* biting, three experimental treatments were set up in the study. Five *B. papyrifera* plants of similar sizes and heights were selected for each experiment group. For the blank treatment group (CK) with no treatment, three leaves were selected from each *B. papyrifera* plant, rinsed with ultrapure water, and collected after air-drying. For the wounding treatment group (positive control, WT), three rinsed leaves of each *B. papyrifera* were clipped to approximately half acreage using scissors after air drying, and these leaves were collected one hour later. For the feeding treatment group (FT), three rinsed leaves of each *B. papyrifera* were bitten by *M. fortis* until more than half the acreage was reached and then collected one hour later. These leaves were immediately frozen in liquid nitrogen, and fifteen leaves in each group were mixed and stored at –80°C for metabolite determination.

The metabolites from leaf samples of different experimental groups were extracted according to the following steps. Firstly, one milliliter of the mixture of organic solvents at a specific volume ratio (methanol:acetonitrile:water = 2:2:1, 20 mg/L) was added to 50 mg of sample in each group and mixed adequately. The sample was ground at 45 Hz for 10 min after adding the steel balls, subjected to ultrasonic treatment for 10 min in an ice bath, and then maintained at –20°C for 1 h. Sample was centrifuged at 4°C and 12,000 rpm for 15 min, and a total amount of 500 µL of supernatant was transferred into EP tubes for each group. The supernatant was dried using a vacuum concentrator, and 160 µL of mixture at a specific volume ratio (acetonitrile:water = 1:1) was added to dried samples. The samples were then mixed for 30 s and subjected to ultrasonic treatment in an ice bath for 10 min. Finally, 120 µL of supernatant was collected and used for determination.

### 2.3 LC-MS/MS analysis

The LC-MS/MS analysis was done based leaf extract. The raw data collected using MassLynx V4.2 was processed by Progenesis QI software for peak extraction, peak alignment, and other data processing operations based on the Progenesis QI software online METLIN database and Biomark’s self-built library for identification. Concurrently, theoretical fragment identification and mass deviation were all within 100 ppm. The original metabolome data were uploaded to a public database (OMIX, China National Center for Bioinformation/Beijing Institute of Genomics, Chinese Academy of Sciences) as described in the data availability statement.

After normalizing the original peak area information to the total peak area, a follow-up analysis was performed. Principal component analysis and Spearman’s correlation analysis were used to assess the repeatability of the samples within the group and the quality control samples. The identified compounds were searched for classification and pathway information using the KEGG (Kanehisa and Goto, 2000) and HMDB (Wishart et al., 2018) databases. Based on the relative expression of samples from different groups, differential metabolites were screened by pairwise comparisons of the three groups. Fold change ≥ 2 or fold change ≤ 0.5 were used as the thresholds for differential metabolites.

The Waters Xevo G2-XS QTOF high-resolution mass spectrometer (Wang et al., 2016) can collect primary and secondary mass spectrometry data in MSe mode under the control of acquisition software (MassLynx V4.2, Waters). In each data acquisition cycle, dual-channel data acquisition was performed simultaneously at both low and high collision energies. The low collision energy was 2V, the high collision energy range was 10–40 V, and the scanning frequency was 0.2 s for a mass spectrum. The parameters of the ESI ion source are indicated as follows: capillary voltage: 2000 V (positive ion mode) or -1500 V (negative ion mode); cone voltage: 30 V; ion source temperature: 150°C; desolvent gas temperature 500°C; backflush gas flow rate: 50 L/h; desolventizing gas flow rate: 800 L/h.

### 2.4 Data statistics

The food intake, foraging frequency and foraging duration were summarized with mean value (Mean ± SE). Nonparametric tests (Wilcoxon test base on two related samples) were used to estimate the differences in foraging frequencies of the two feed cages, and an independent samples t-test was used to evaluate the differences in food intake and foraging duration. The p-value of 0.05 was used as the criterion for judging significance. The statistics analysis was implemented using SPSS 18.0 and the figure was drew by Origin 2023.

## 3 Results and analysis

### 3.1 The influence of gender on foraging behaviors of *M. fortis*

The blank control (model 1) and the smell models (model 2) were used to confirm if the direction and smell of the plants influenced the foraging behaviors of *M. fortis*. Sexual comparisons were firstly inspected for these foraging characteristics in the two models (**Supplementary Figure S2**). The only significant difference (P<0.05) was found in comparison of food intakes of BF on the left site in blank control model, while other values were similar in two models between male and female voles (P>0.05). This indicated that the direction and smell of food hardly influenced the feeding preference of *M. fortis*.

**Figure 2:**
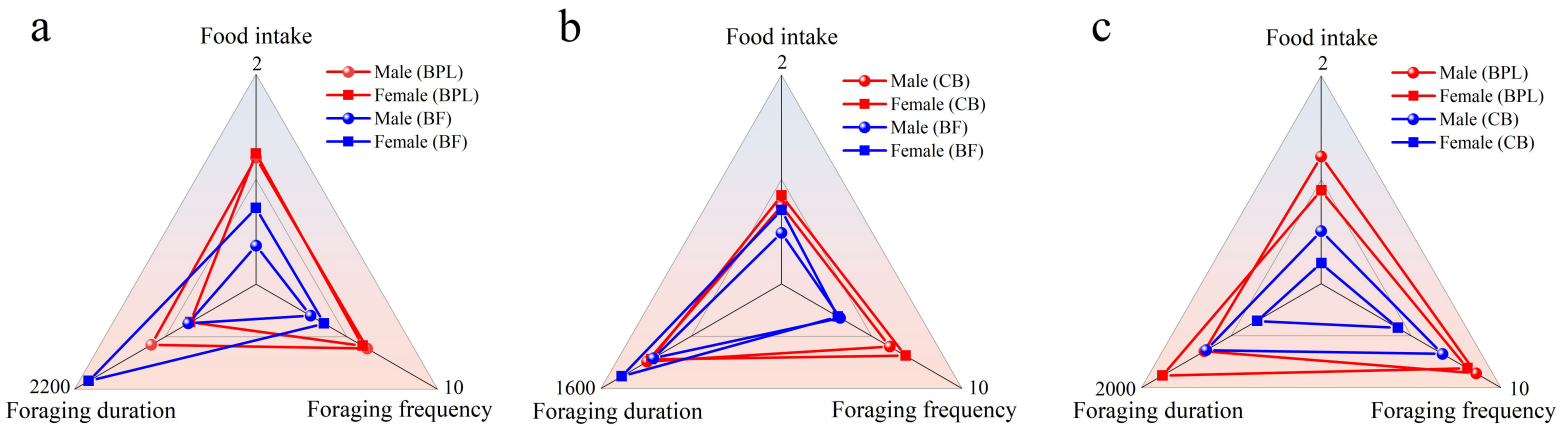
The comparison on foraging behaviors between different gender of *M. fortis* in **(A)** BPL vs BF model, **(B)** CB vs BF model and **(C)** BPL vs CB model.

Foraging characteristics were analyzed and differences were compared between male and female (**Figure 2**). Overall, there were few significant differences between different genders. In BPL vs BF model, foraging duration of female feed on BF was significant at P<0.01 longer than that of male (**Figure 2A**). The male characteristics were similar with female in CB vs BF model, and showed no significant difference (**Figure 2B**). The significant differences (P<0.05) were found in food intake and foraging duration between male and female feed on CB in the BPL vs CB model (**Figure 2C**). Other comparisons showed no significant difference between genders in three models (P>0.05), thus, subsequent analyses were tended to on the base of combined data of all individuals.

### 3.2 Feeding preference of *M. fortis* to *B. papyrifera* leaves

According to the combined data of genders, there were no significant differences in food intake, foraging frequency, or foraging duration between difference cages in blank control, and smell model (**Supplementary Figure S3**). The blank control model demonstrated that the direction of the food hardly influenced their feeding choice. Additionally, it has demonstrated that food intake, foraging frequency, and duration of *M. fortis* attracted by BPL smell were not significant (P>0.05) compared to CB smell. Therefore, the smell of plant foods also exerted no obvious influence on the foraging of *M. fortis*, and this was beneficial for further comparison among BPL, CB and BF with respect to feeding.

**Figure 3:**
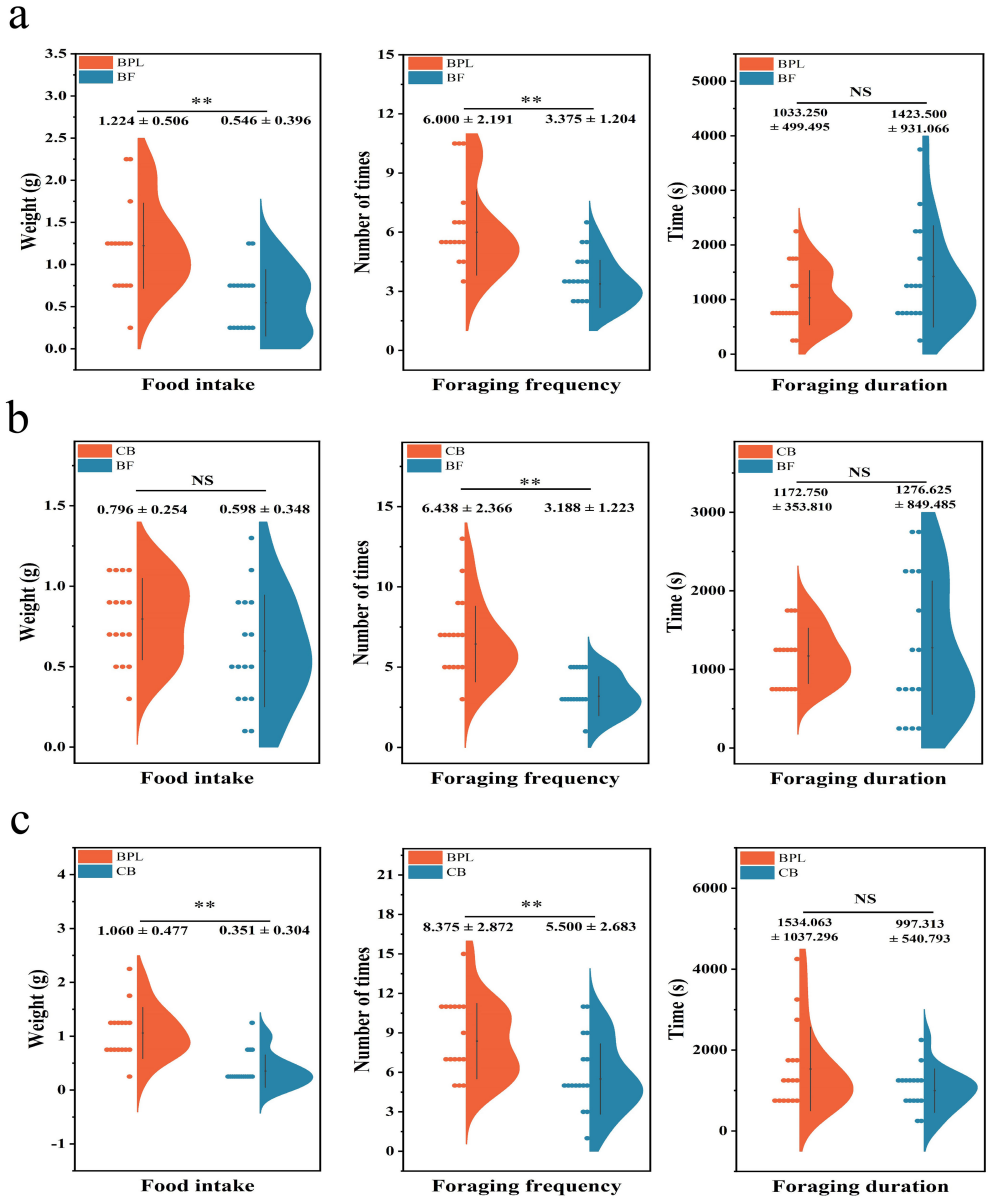
The comparison of feeding preference of *Microtus fortis* **(A)** between BPL VS BF **(B)** between CB VS BF, **(C)** BPL VS CB. Dots on the left site describe distribution of samples, colorful places show the levels of parameters, NS represent P>0.05, ** represent P<0.01.

Base on a comparison between BPL and BF, *M. fortis* preferred to feed on BPL (**Figure 3A**). The food intake of BPL was 1.22 ± 0.51 g, and it exhibited the difference with high level of significance compared to BF (P<0.01) where the food intake was just 0.55 ± 0.40 g. An significant difference at P<0.01 in foraging frequency was also observed between BPL and BF, where the frequency of BPL was 6.00 ± 2.20 and that of BF was 3.38 ± 1.20. These results indicated that *M. fortis* tended to forage and feed more on BPL than they did on BF. Although the foraging duration of BPL was less than that of BF, the difference was not significant (P>0.05).

The CB and BF models demonstrated a slightly greater attraction of *M. fortis* to CB than to BF (**Figure 3B**). There was no significant difference in food intake (P>0.05), but consumption of CB (0.80 ± 0.25 g) was higher than BF (0.60 ± 0.35 g). Meanwhile, the foraging frequency of CB was 6.44 ± 2.37, which was higher than 3.188 ± 1.233 for BF. Foraging duration was not significantly different between CB and BF, and the levels were similar, although the basal fodder range was wider.

The last comparison was BPL and the CB model, and it was demonstrated that BPL was more attractive to *M. fortis* (**Figure 3C**). Food intake, foraging frequency, and foraging duration of BPL were higher than those for CB. Food intake and foraging frequency of BPL were 1.06 ± 0.487 g and 8.38 ± 2.87, and both were much higher than CB (P<0.01) which were 0.35 ± 0.30 g and 5.50 ± 2.68. Although there was no significant difference in foraging duration (P>0.05), the BPL duration was longer than that for CB. The feeding experiment demonstrated that plant food attracted *M. fortis* more on feeding than did basal fodder, while BPL exhibited the highest attraction among the three types of food.

Statistical comparisons were also conducted separately for males and females to verify the trend (**Supplementary Figure S4**), and indicated that most parameters were consistent with the results shown in **Figure 3**. However, the foraging duration for males and females in BPL vs BF differed significantly. The combined data could support the comparison of the feeding preference of *M. fortis* towards different foods.

### 3.3 Identification of metabolites in *B. papyrifera* leaves

**Figure 4:**
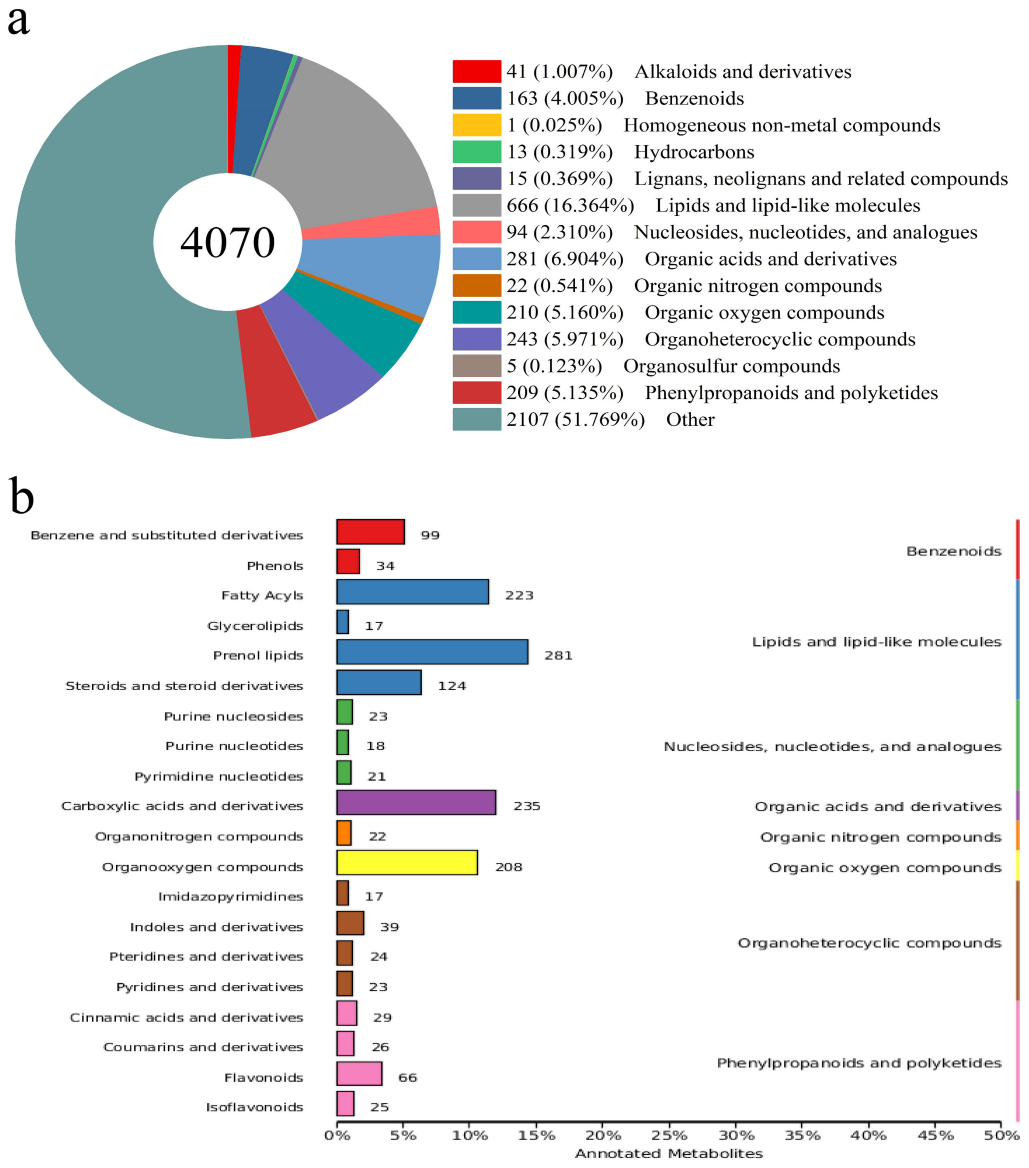
The annotation of metabolites in *Broussonetia papyrifera* leave **(A)** amount and classification of metabolites, **(B)** secondary classification of metabolites.

Primary metabolites such as lipids are the main substances in BPL. A total of 4,070 metabolites were detected in BPL, and nearly half were classified into thirteen categories (**Figure 4A**), including 666 lipids and lipid-like molecules, 281 organic acids and derivatives, and 243 organo heterocyclic compounds. According to secondary classification, 223 fatty acyls and 281 prenol lipids belonged to the lipids and lipids-like molecules category (**Figure 4B**). At this grade, the familiar secondary metabolites were annotated, and 34 phenols belonged to the benzenoids category, while 66 flavonoids and 25 isoflavonoids belonged to the phenylpropanoids and polyketides category.

### 3.4 Differential metabolites among different treatments

**Figure 5:**
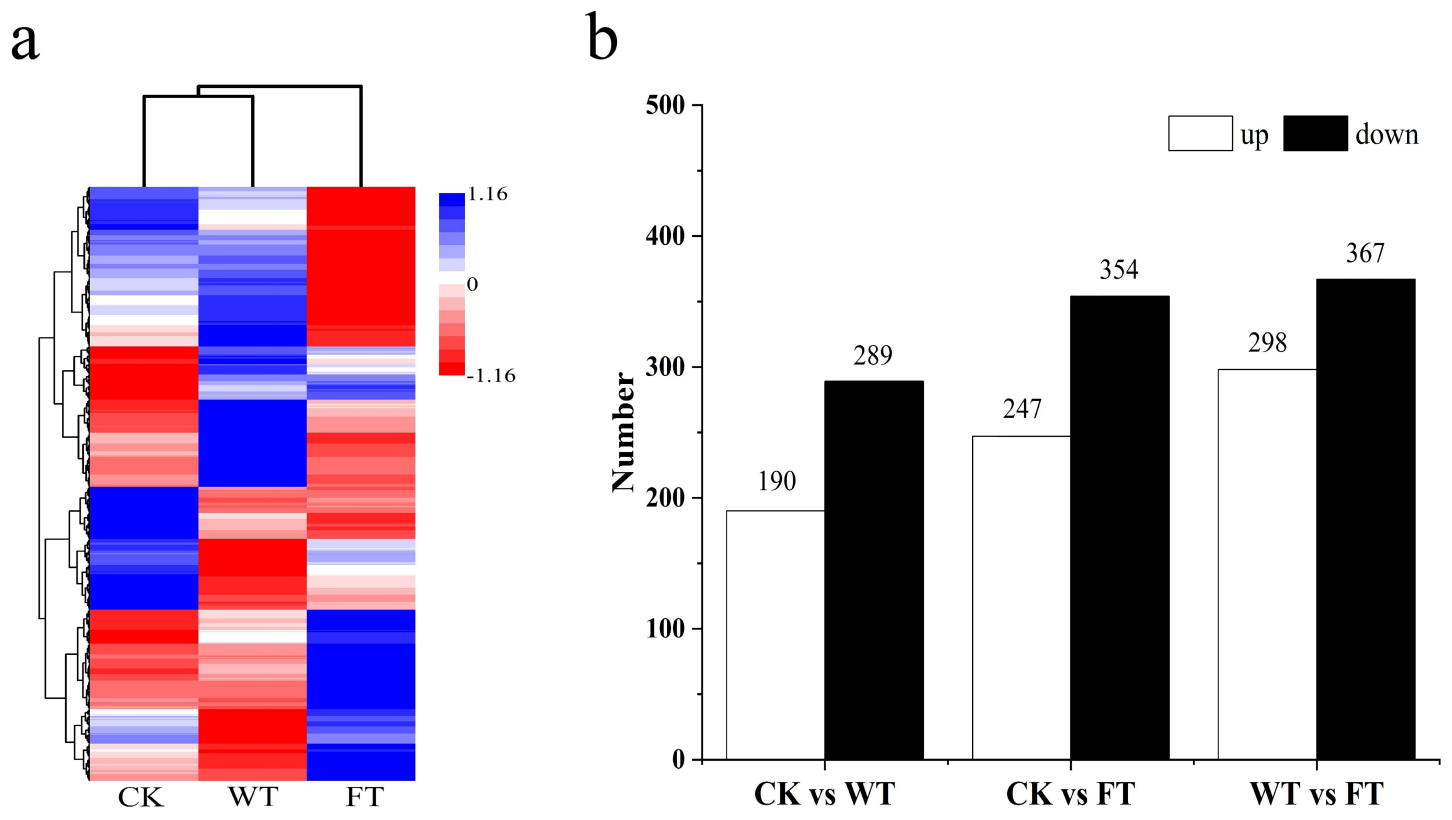
The information of metabolites in different forms of *Broussonetia papyrifera* leaves **(A)** cluster analysis of different groups, **(B)** amount differential metabolites in each comparison.

A metabolite cluster tree was constructed according to the correlation of expression levels among metabolites, with one branch of the tree corresponding to the metabolite clusters whose relative expression levels were highly correlated. The tree indicated that CK and WT were categorized into one branch, whereas FT was categorized onto its own branch (**Figure 5A**). This implies that the variation in substances in the BPL was more significant after experiencing bites from *M. fortis*. Based on the relative expression of metabolites from BPL in the different groups, 479, 601, and 665 differential metabolites were observed between CK and WT, CK and FT, and WT and FT, respectively (**Figure 5B**). The number of down-regulated metabolites in the three comparisons was greater than that of the up regulated metabolites.

The top 10 up-regulated and down-regulated metabolites in different comparisons are presented in **Table 1**. Three substances were significantly regulated in all comparisons including 6beta,7beta-Dihydroxykaurenoic acid, 6’’-O-Malonyldaidzin, and brassinolide. It was observed that 6beta,7beta-Dihydroxykaurenoic acid was up-regulated in CK vs. WT and CK vs. FT, but it was down-regulated in WT vs. FT. Four differential metabolites, including nystatin, obacunone, (22R,23R)-22,23-Dihydroxy-campest-4-en-3-one, and brassicasterol were regulated significantly only in the top ten of CK vs. FT.

**Table 1:** Top 10 regulated metabolites in comparisons between different *Broussonetia papyrifera* leaves.

CK vs WT	CK vs FT	WT vs FT	Type
6beta,7beta-Dihydroxykaurenoic acid	6’’-O-Malonyldaidzin	Desmosterol	Up
Glucoputranjivin	Glucoputranjivin	L-Dopachrome	Up
6’’-O-Malonyldaidzin	6beta,7beta-Dihydroxykaurenoic acid	3-Hydroxyisovaleric acid	Up
Brassinolide	Budesonide	Indole-3-methanamine	Up
Arginyl-Isoleucine	Arginyl-Isoleucine	Budesonide	Up
2-(Formamido)-N1-(5’-phosphoribosyl) acetamidine	1,3-Digamma Linolenin	Threoninyl-Leucine	Up
Ginsenoside F1	Nystatin	Hydroxyanigorufone	Up
Ginsenoside Rh3	Adrenochrome	Cafestol	Up
1,3-Digamma Linolenin	Obacunone	Adrenochrome	Up
1-(9Z,12Z-octadecadienoyl)-2-(5Z,8Z,11Z,14Z-eicosatetraenoyl)-sn-glycerol	(22R,23R)-22,23-Dihydroxy-campest-4-en-3-one	6’’-O-Malonyldaidzin	Up
Lupanine	Lupanine	Ethyl phenylglycidate	Down
3-Hydroxyisovaleric acid	Ethyl phenylglycidate	L-NIO	Down
Desmosterol	(S)-5-Amino-3-oxohexanoic acid	(S)-5-Amino-3-oxohexanoic acid	Down
L-Dopachrome	L-NIO	Brassinolide	Down
Indole-3-methanamine	Linoleoyl ethanolamide	Thiamin monophosphate	Down
Hydroxyanigorufone	Thiamin monophosphate	Ginsenoside Rh3	Down
Linoleoyl ethanolamide	Brassicasterol	6beta,7beta-Dihydroxykaurenoic acid	Down
Soyasaponin Ba	Brassinolide	2-Methyl-1-hydroxypropyl-ThPP	Down
Dehydrotomatine	2-Methyl-1-hydroxypropyl-ThPP	Hydroquinine	Down
Naringin	13-Dihydrocarminomycin	I-Urobilinogen	Down

### 3.5 Excavation of responsive substances in *B. papyrifera* leaves

**Figure 6:**
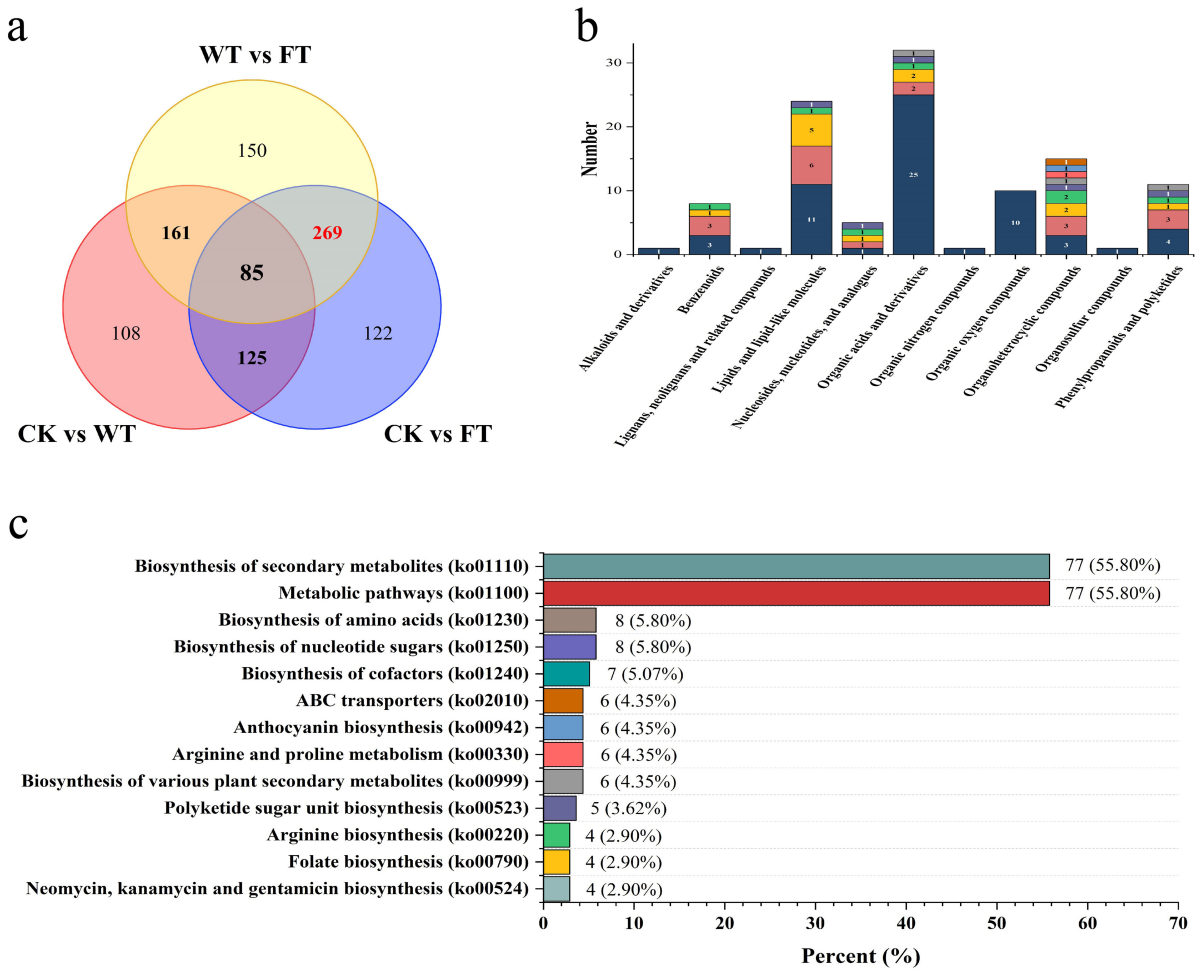
Information of screened metabolites **(A)** Venn diagram of differential metabolites among comparisons, **(B)** The classification of the screened differential metabolites, different colors mean quantity of secondary categories, **(C)** KEGG classification of screened differential metabolites.

The Venn diagram indicates approximately 85 common differential metabolites among the three comparisons (**Figure 6A**). The numbers of unique differential metabolites in CK vs. WT, CK vs. FT, and WT vs. FT were 108, 122, and 150, respectively. The intervention with WT leaves was aimed at avoiding substances related to the self-curing strategy of *B. papyrifera*. Therefore, potential substances in the BPL that respond to the bite of *M. fortis* should be excavated from the 269 overlapping metabolites between CK vs. FT and WT vs. FT, thus indicating that these metabolites of FT were significantly different from those of CK and WT.

Among the 269 metabolites, approximately 109 were annotated in the HMDB database and identified as 40 substances classified into 11 categories (**Figure 6B**). There were 32 metabolites belonging to six types of organic acids, and their derivatives were the most abundant. Lipids and lipid-like molecules were the second most abundant category, containing 24 metabolites belonging to six different types. Focusing on secondary metabolites, there were five types of phenylpropanoids and polyketides, including four flavonoids and three isoflavonoids. There were four types of benzenoids, including three phenols and one anthracene, and there was only one tropane alkaloid and derivative.

According to the KEGG annotation results, 138 of the 269 metabolites were identified in 83 KEGG pathways (**Figure 6C**). Approximately 13 pathways annotated more than three substances, and of these, the biosynthesis of secondary metabolites and metabolic pathways annotated 77 substances, both of which were higher than 50%. Additionally, the biosynthesis of various PSMs annotated six substances, and most of the targeted metabolites were related to PSMs.

Pathways involving the biosynthesis of specific PSMs such as terpenoids, flavonoids, alkaloids, and 24 other substances were screened (**Table 2**). There were 7 metabolites whose difference of relative expression between CK and WT were within 10%, including geranylgeranyl diphosphate, 3-Hydroxyindolin-2-one, gaccatin III, loganate, kaempferol 3-O-beta-D-glucosyl-(1≥2)-beta-D-glucoside, medicarpin, and gibberellin A14. These substances are involved in the biosynthesis of diterpenoids, ubiquinones, benzoxazinoids, monoterpenoids, isoflavonoids, flavones, and flavonols. Remarkably, geranylgeranyl diphosphate was involved in three biosynthesis pathways, and its relative expression was much lower than that in CK and WT.

**Table 2:** The relative expression and KEGG annotation of substances related to biosynthesis of secondary metabolites.

Metabolite	FC			Pathway
	WT/CK	FT/CK	FT/WT	
Geranylgeranyl diphosphate	0.94	0.40	0.42	Biosynthesis of secondary metabolites (ko01110) Biosynthesis of various plant secondary metabolites (ko00999) Diterpenoid biosynthesis (ko00904) Ubiquinone and other terpenoid-quinone biosynthesis (ko00130) Terpenoid backbone biosynthesis (ko00900)
Hesperetin 7-O-glucoside	0.90	0.36	0.40	Flavonoid biosynthesis (ko00941)
Narcotoline hemiacetal	0.75	0.28	0.37	Biosynthesis of secondary metabolites (ko01110) Isoquinoline alkaloid biosynthesis (ko00950)
Asperuloside	1.39	0.28	0.20	Biosynthesis of secondary metabolites (ko01110) Monoterpenoid biosynthesis (ko00902)
Shikonin	0.52	0.21	0.40	Biosynthesis of secondary metabolites (ko01110) Ubiquinone and other terpenoid-quinone biosynthesis(ko00130)
Kaempferol 3-sophorotrioside	1.16	0.33	0.28	Biosynthesis of secondary metabolites (ko01110) Flavone and flavonol biosynthesis (ko00944)
Catharanthine	0.87	0.29	0.33	Biosynthesis of secondary metabolites (ko01110) Indole alkaloid biosynthesis (ko00901)
Glycitin	0.83	0.37	0.44	Isoflavonoid biosynthesis (ko00943)
Solavetivol	1.12	2.45	2.20	Biosynthesis of secondary metabolites (ko01110) Sesquiterpenoid and triterpenoid biosynthesis (ko00909)
3-Hydroxyindolin-2-one	1.02	0.47	0.46	Biosynthesis of secondary metabolites (ko01110) Biosynthesis of various plant secondary metabolites (ko00999) Benzoxazinoid biosynthesis (ko00402)
Ecgonine	1.47	0.41	0.28	Tropane, piperidine and pyridine alkaloid biosynthesis (ko00960)
Baccatin III	0.98	0.45	0.46	Biosynthesis of secondary metabolites (ko01110) Diterpenoid biosynthesis (ko00904)
Pelargonidin	1.46	0.47	0.32	Biosynthesis of secondary metabolites (ko01110) Flavonoid biosynthesis (ko00941)
Berbamunine	0.99	2.69	2.70	Biosynthesis of secondary metabolites (ko01110) Isoquinoline alkaloid biosynthesis (ko00950)
Loganate	0.70	0.34	0.49	Biosynthesis of secondary metabolites (ko01110) Monoterpenoid biosynthesis (ko00902)

## 4 Discussion

### 4.1 Potential of *B. papyrifera* for plant rodenticide

Using chemical rodenticides for conservation and use in agriculture remains important; however, they are acute or quick-acting and lethal, causing subsequent environmental concerns and leading to the urgent need for developing moderate inhibitors. Based on animal–plant interactions, compounds or chemicals derived from natural sources have inspired many effective medicines and biocides, including rodenticides and vertebrate pesticides. Natural compounds have evolved due to various pressures and remain a source for developing new drugs and biocides (Eason, 2018). In recent years, efforts have been made towards using plant compounds as botanical insecticides (Amoabeng et al., 2019). Green inhibitors can be a good alternative to chemical drugs and can be utilized to control pests and minimize ecological losses. Different strategies for plants to cope with being eaten can lead to different types of rodenticides, including refusal to eat, poisoning, and infertility. Many candidate plant species could be rapidly developed into new products, and the emphasis on plant protection has shifted from lethal chemical substances to integrated pest management.

Palatability and the effectiveness are the two most important factors in developing plant-based rodenticides. In the present study, we compared the feeding preference of *M. fortis* for *B. papyrifera* leaves compared to that for their primary foods. The experiment referred to the cafeteria measurement model (Shafat et al., 2009) that could accurately detect the food choices of animals during a regular period, and we also utilized video to avoid environmental interference. Feeding strategies for animals are based on genetic messages and personal foraging experiences (Velázquez-Martínez et al., 2010; Provenza et al., 2003; Owen, 1992), and the feed pattern of offspring primarily results from the parental generation (Heinsohn, 1991). The *M. fortis* individuals in this study had learned to feed on basal fodder since birth. The optimal recipe model assumes that abundant suitable foods lead animals to gradually feed on a single food (Sun, 2001).The basal fodder contained 20% crude protein, 4.8% crude fat, and 17.1% caloric value (Zhu et al., 2011), and this was regarded as the single food that maintains the growth of voles. Thus, *B. papyrifera* leaves and *C. brevicuspis* are challenged when they suddenly become food resources for *M. fortis*. However, the voles immediately changed their food preference, supporting the voles’ feed strategy of adjusting to gainable *B. papyrifera* leaves and *C. brevicuspis*. Certain species will alter their food choice under different physiological conditions (Newman et al., 1994; Wang and Provenza, 1996), and one study demonstrated that *M. fortis* increased food intake but did not change their food choice. This supported the accuracy of our comparison. The smell of food may influence the foraging choice of animals (Moelzner and Fink, 2014). According to the smell experiment, the voles exhibited insignificant differences in foraging frequency and duration, thus indicating that the smell of the two plants may not be an essential factor in the feeding of voles. The feeding experiment indicated that *M. fortis* prefer to feed on *B. papyrifera* leaves rather than on *C. brevicuspis* or basal fodder under the micro-environment. This suggested that *B. papyrifera* leaves have good palatability for *M. fortis*, satisfying a precondition of plant rodenticides.

Various plant substances have antifertility activities that disrupt normal reproductive functions (Daniyal and Akram, 2015; Mali et al., 2002). When we previously fed *M. fortis* with *B. papyrifera* leaves, the development of their sexual organs was inhibited, and they showed lower fertility. That indicated that a component in the leaves could restrain the reproduction of *M. fortis* (Wang et al., 2023). This combined with the results from the present study, suggests *B. papyrifera* leaves have good palatability for the voles, which supports that *B. papyrifera* has a strong possibility to be a plant rodenticide resource.

### 4.2 Effective substances can be acquired from *B. papyrifera* leaves base on its rapid response

Animal–plant interactions are important components and processes in ecosystems and are key relationships in the food web (Elsayed and Din, 2019; Schoener, 1974). Foraging is an essential behavior for the survival and reproduction of animals (Davidson and El Hady, 2019). Animals adapt their feeding frequency based on the obtainability, palatability, and nutritive value of plants (Rutter, 2006). Herbivores would prefer to feed on plants with high protein and energy (Wang and Chen, 2012; Provenza et al., 2003). Generally, animals are resourceful and use advanced feeding strategies. However, plants develop a series of physical and chemical defense mechanisms in response to animal feeding (Yuan et al., 2009). As the primary producers in the ecosystem, plants have evolved effective mechanisms against animals. One of the aims of our study was to identify the responsive substance of *B. papyrifera* leaves to bites from *M. fortis*, which may be potentially helpful in rodent management. The active substances of plants exhibit different functions. Their primary metabolites affect plant growth, whereas the secondary metabolites they produce are used for defense (Bennett and Wallsgrove, 1994). Various groups of plant substances, such as alkaloids, steroids, terpenoids, essential oils and phenolics, have inhibitive effects on herbivores (Shaalan et al., 2005). These substances can be isolated using different extraction methods and could be used to rapidly produce the related inhibitors. For example, pine needle oil was used to repel *Geomys bursarius* from gnawing on cables (Epple et al., 2001), and *Apodemus sylvaticus* was deterred from consuming valuable tree seeds by using capsaicin (Willoughby et al., 2011).

According to the metabolome data, there were numerous lipids and acids that organisms needed, and this may have led to the preference by *M. fortis*. Undeniably, the functions of substances are extensive, and confirming the various functions requires long-term work. The *B. papyrifera* leaves contain several secondary metabolites, like phenols, steroids, flavonoids and isoflavonoids; most are toxic to herbivores or inhibit their development (Hanley et al., 2007). Additionally, significant changes regarding the metabolites occurred in the leaves after different damages. In fact, several substances are involved in the self-healing mechanism of plants after physical damage (Yuan et al., 2009). This prompted us to intervene in the wounding treatment of leaves to prevent the synthesis of these substances as much as possible. The defense mechanisms of plants typically form over a short period, and a study has revealed that the saliva of herbivores can stimulate these defense mechanisms (Arimura, 2021). The results revealed that the variation in metabolites in *B. papyrifera* leaves was more significant after being bitten by *M. fortis* for a short period, and 269 substances related to defense were screened. Although most of these substances were primary metabolites, we also observed some defensive secondary metabolites, such as tannins that can restrict nutrient digestibility and utilization by animals (Tiku, 2018) and alkaloids that exert toxicological effects in animals (Feng et al., 2022). Moreover, more than half of the targeted metabolites were annotated in the biosynthesis of various secondary metabolites that are the primary defensive substances in plants, such as alkaloids, which do not have a primary function in plants. However, many are toxic to animals, vertebrates and arthropods, and terpenoids and flavonoids also contribute to both direct and indirect defenses (Li and Liu, 2002). Monoterpene, a terpenoid, is poisonous to animals and can disturb the energy metabolism of animals after they feed on plants containing monoterpenes (Boyle and Dearing, 2003). Therefore, some targeted metabolites in *B. papyrifera* leaves may respond to bites from *M. fortis*. In almost all cases, upon herbivore attack, an inducible defense is established locally on the site of infestation as well as systemically throughout the whole plant, albeit in some cases with lower intensities (Mithöfer and Boland, 2012). For example, vibrations associated with herbivore chewing can induce chemical defenses in Arabidopsis, even in sites far from the wounding site (Appel and Cocroft, 2014). This research is particularly intriguing as it focused on the unique ability of *B. papyrifera* to produce a variety of defensive substances in response to the bite of *M. fortis*. This rapid response suggested a potential use of the plant in the production of plant-derived rodenticides.

### 4.3 Limitations and expectations of application based on the broader ecosystem

Optimal nutrient acquisition by animals is a dynamic process. They alter their feeding strategy when food resources or the environment change (Raubenheimer and Jones, 2006). The higher nutritional value of *B. papyrifera* leaves provided stronger attraction for *M. fortis*. Nevertheless, the generalizability of the findings is limited by the narrow scope and we also need to consider the material changes in the leaves of *B. papyrifera* to determine the appropriate period. Recognizing the limitations of this study is beneficial for further explorations, and these projects are relevant to the current limitation. Moreover, an important point is that rodents other than *M. fortis*, such as *Apodemus agrarius*, cause damage to crops around Dongting Lake wetland (He et al., 2023). Whether *B. papyrifera* leaves have good palatability to other rodents and how the leaves respond to their respective bite is unknown. Wild *B. papyrifera* leaves are used to feed livestock but show poor palatability for pigs and cattle due to the abundant secondary metabolites. Hence, the hybrid *B. papyrifera* was developed to solve this problem. Whether different *B. papyrifera* will have a similar potential regarding rodenticide manufacturing, is a concern. We hypothesized that the substances responding to bites from *M. fortis* might involve the composite rodenticide used in areas sustaining pest damage. Our comparison aimed to reduce the range of defense molecules, and a series of studies should be conducted to confirm the specific effective substances and verify the extract. Verifying these hypotheses may help us determine the universal defensive substances of *B. papyrifera* leaves.

This study put forward the potential of *B. papyrifera* leaves as a plant resource to be used to produce rodent rodenticides. The plant-derived sterile inhibitors are not as lethal as rodenticides such as anticoagulants. Additionally, its continuous management may rely on the feed behavior of rodents. Rodents can exploit a wide variety of food. This requires that the potential plant inhibitors have great palatability and even have specific advantage. A series of studies should explore various rodents and the specific eco-environment to support the generalizability to a broader ecological context.

## 5 Conclusion

In this study, we compared the food intake, foraging frequency and duration of *M. fortis* on *B. papyrifera* leaves, *C. brevicuspis*, and daily fodder. We analyzed the potential defense substances of *B. papyrifera* leaves in response to bites from *M. fortis*. *B. papyrifera* leaves exhibited the strongest attraction to the voles during feeding. They preferred to forage and feed on *B. papyrifera* leaves more than on the other two, suggesting good palatability of the leaves to rodents. A total of 4,070 substances were detected in *B. papyrifera* leaves, and based on the relative expression of these substances, bitten leaves were significantly different from comparative leaves. Through the analysis of differential metabolites, we screened for substances that may respond to bites from *M. fortis*, including medicarpin, loganate, and geranylgeranyl diphosphate. These substances instantly contribute to the biosynthesis of secondary metabolites which are the primary substances used to against animals. This study demonstrated the relationship between *B. papyrifera* leaves and *M. fortis*, and put forward that *B. papyrifera* leaves can be used to develop plant rodenticides. This will be useful for further research examining the interaction between plants and herbivores as well as the resource utilisation of *B. papyrifera* and the management of rodent pests.

## Data Availability

The datasets presented in this study can be found in online repositories. The names of the repository/repositories and accession number(s) can be found below: OMIX, China National Center for Bioinformation/Beijing Institute of Genomics, Chinese Academy of Sciences (https://ngdc.cncb.ac.cn/omix: accession no. OMIX005478, PRJCA022356).

## Supplementary material

Supplementary Figure 1 The structure of apparatus and design of models in feeding experiment. *1. There were four male and four female voles used in all models. 2. Each vole was tested twice in each model, the locations of two food trays were exchanged during the second observation. Besides, the bottles in model 2 were also exchanged. 3. Before one’s observation, all apparatus should be washed and disinfected to erase the smell and trace of previous vole. 4. The experiment using a camera that was 1 m above the apparatus that allowed the full view, when vole was put into drink cage, then stated videoing for 90 minutes once.

Supplementary Figure 2 The comparison on foraging behaviors between different gender of *M. fortis* in **(A)** blank control model, **(B)** BPL smell vs CB smell model.

Supplementary Figure 3 The comparison of feeding preference of *Microtus fortis* **(A)** between BF1 vs BF2 **(B)** between BPL smell vs CB smell.

Supplementary Figure 4 The comparison of feeding preference of *Microtus fortis* for males and females among BPL, CB and BF.
