# Supplementary figures and images for: The feeding preference and bite response between Microtus fortis and Broussonetia papyrifera
Source: Front Plant Sci. 2024 Sep 9;15:1361311. doi: 10.3389/fpls.2024.1361311 (PMC11417685; doi:10.3389/fpls.2024.1361311)

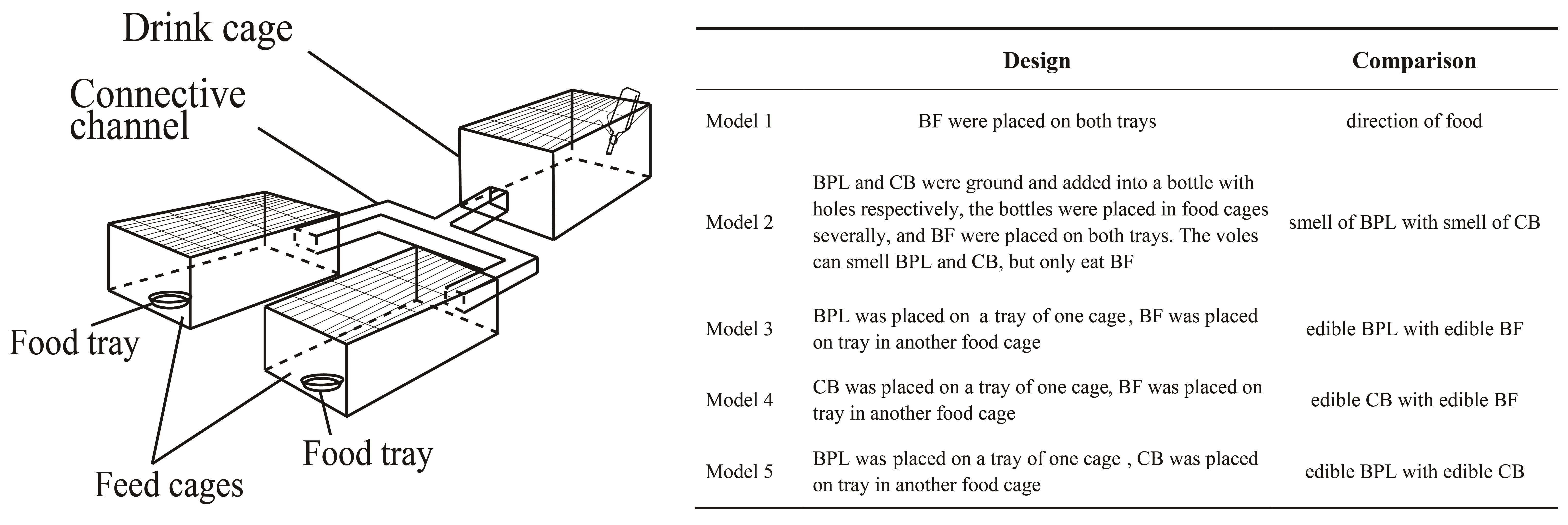

Supplement: Supplementary Figure 1 — The structure of apparatus and design of models in feeding experiment. *1. There were four male and four female voles used in all models. 2. Each vole was tested twice in each model, the locations of two food trays were exchanged during the second observation. Besides, the bottles in model 2 were also exchanged. 3. Before one’s observation, all apparatus should be washed and disinfected to erase the smell and trace of previous vole. 4. The experiment using a camera that was 1 m above the apparatus that allowed the full view, when vole was put into drink cage, then stated videoing for 90 minutes once. [file DataSheet1.zip › Supplementary files/Figure S1.jpg]

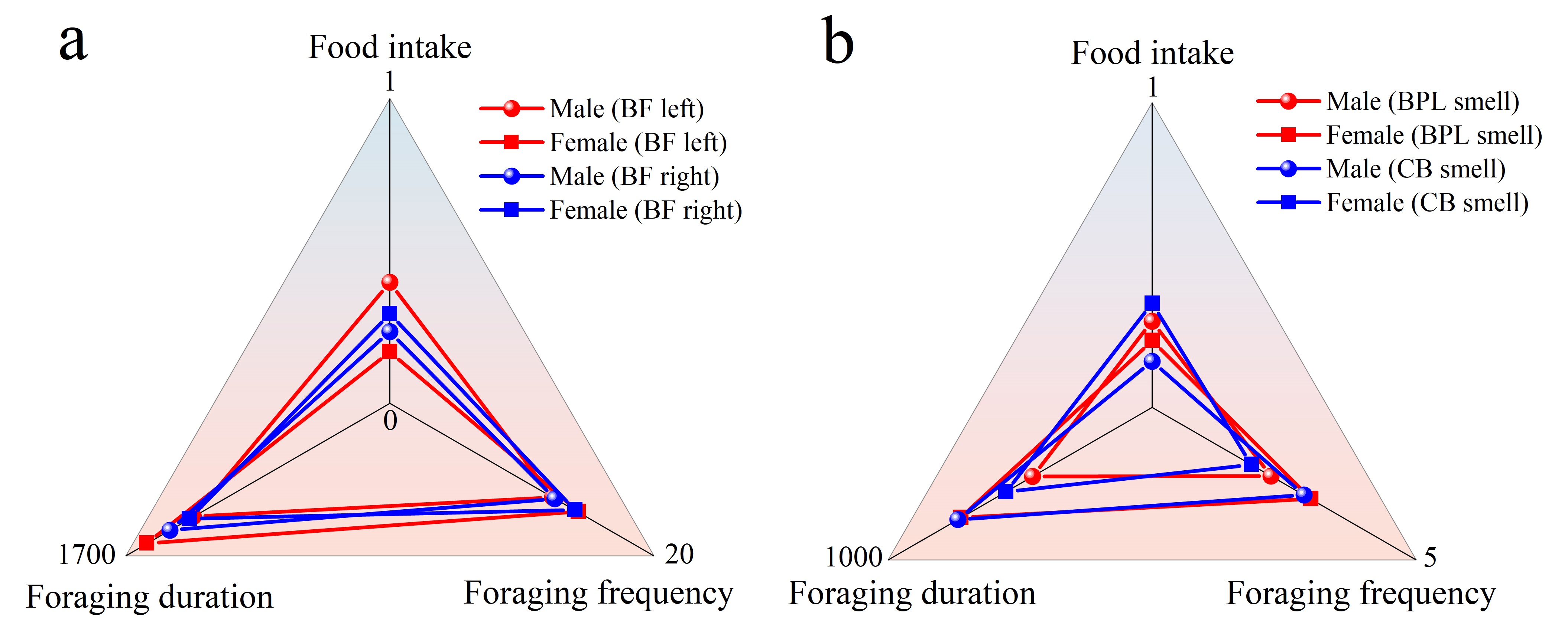

Supplement: Supplementary Figure 1 — The structure of apparatus and design of models in feeding experiment. *1. There were four male and four female voles used in all models. 2. Each vole was tested twice in each model, the locations of two food trays were exchanged during the second observation. Besides, the bottles in model 2 were also exchanged. 3. Before one’s observation, all apparatus should be washed and disinfected to erase the smell and trace of previous vole. 4. The experiment using a camera that was 1 m above the apparatus that allowed the full view, when vole was put into drink cage, then stated videoing for 90 minutes once. [file DataSheet1.zip › Supplementary files/Figure S2.jpg]

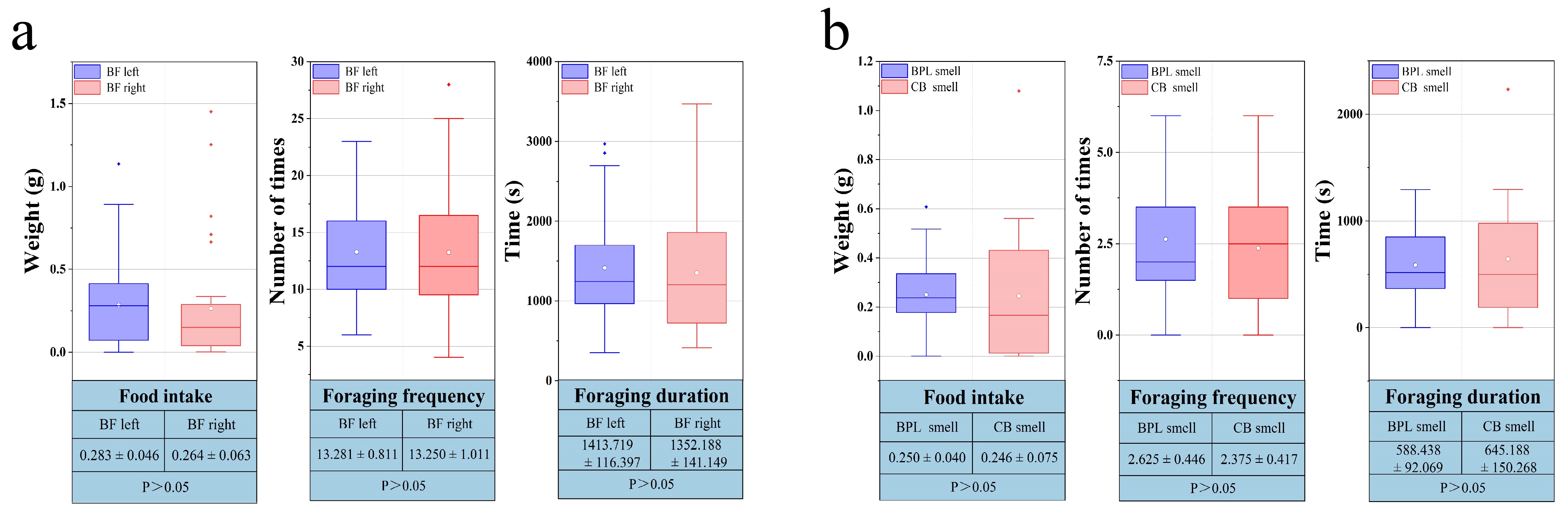

Supplement: Supplementary Figure 1 — The structure of apparatus and design of models in feeding experiment. *1. There were four male and four female voles used in all models. 2. Each vole was tested twice in each model, the locations of two food trays were exchanged during the second observation. Besides, the bottles in model 2 were also exchanged. 3. Before one’s observation, all apparatus should be washed and disinfected to erase the smell and trace of previous vole. 4. The experiment using a camera that was 1 m above the apparatus that allowed the full view, when vole was put into drink cage, then stated videoing for 90 minutes once. [file DataSheet1.zip › Supplementary files/Figure S3.jpg]

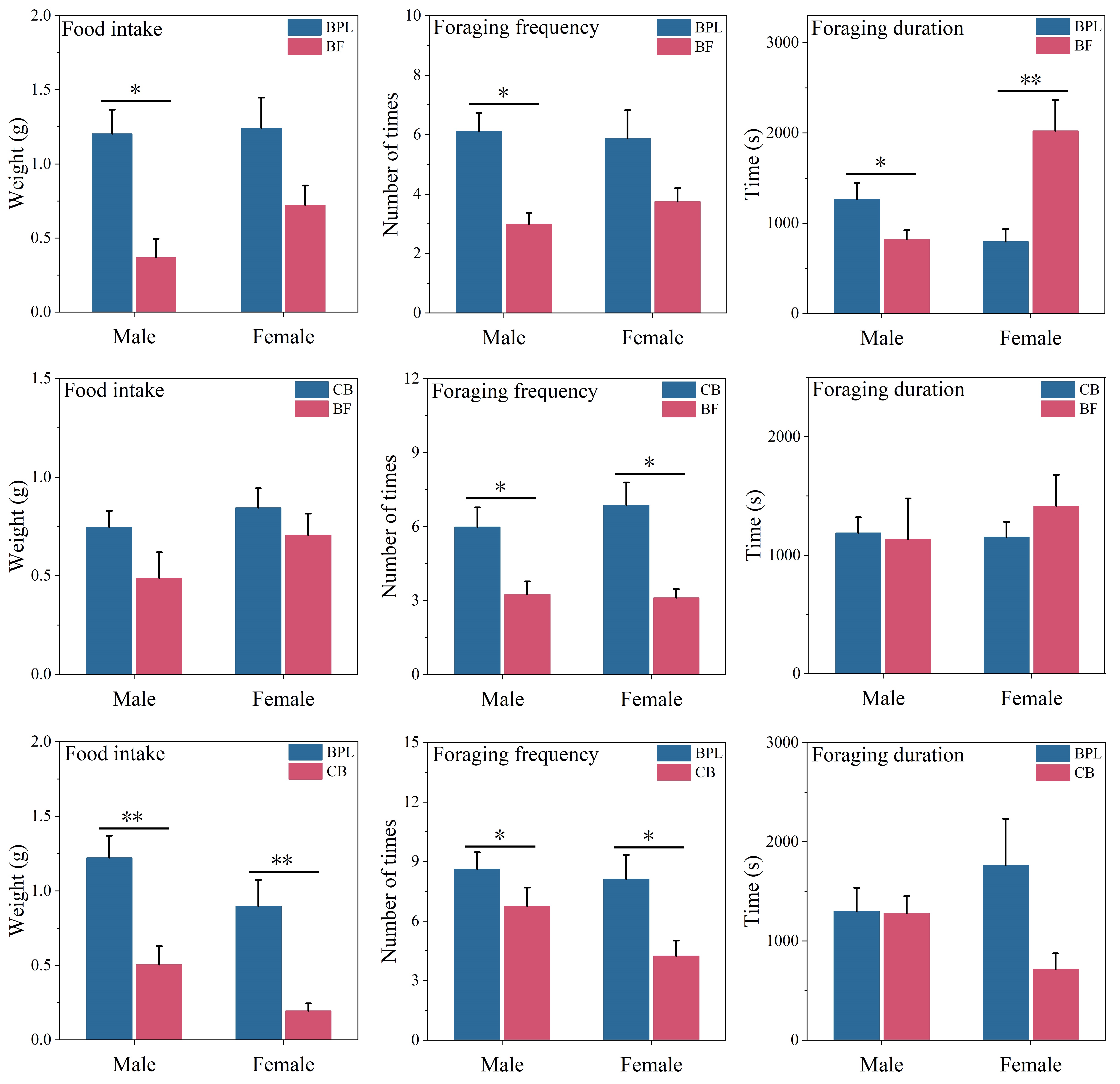

Supplement: Supplementary Figure 1 — The structure of apparatus and design of models in feeding experiment. *1. There were four male and four female voles used in all models. 2. Each vole was tested twice in each model, the locations of two food trays were exchanged during the second observation. Besides, the bottles in model 2 were also exchanged. 3. Before one’s observation, all apparatus should be washed and disinfected to erase the smell and trace of previous vole. 4. The experiment using a camera that was 1 m above the apparatus that allowed the full view, when vole was put into drink cage, then stated videoing for 90 minutes once. [file DataSheet1.zip › Supplementary files/Figure S4.jpg]
